# L-arginine combination with 5-fluorouracil inhibit hepatocellular carcinoma cells through suppressing iNOS/NO/AKT-mediated glycolysis

**DOI:** 10.3389/fphar.2024.1391636

**Published:** 2024-05-22

**Authors:** Yile Hu, Yihao Xing, Gaolu Fan, Huaxia Xie, Qingzan Zhao, Ling Liu

**Affiliations:** ^1^ College of Basic Medicine and Forensic Medicine, Henan University of Science and Technology, Luoyang, China; ^2^ Department of Pharmacy, Luoyang Third People’ Hospital, Luoyang, China; ^3^ School of Basic Medical Sciences, Zhengzhou University, Zhengzhou, China

**Keywords:** aerobic glycolysis, L-arginine, nitric oxide, inducible nitric oxide synthase, 5-fluorouracil

## Abstract

L-arginine can produce nitric oxide (NO) under the action of inducible nitric oxide synthase (iNOS), while 5-fluorouracil (5-FU) can induce the increase of iNOS expression. The present study was to investigate the mechanism of L-arginine combined with 5-FU regulating glucose metabolism of hepatocellular carcinoma (HCC) through iNOS/NO/AKT pathway. The combination of L-arginine and 5-FU resulted in decreased cell survival and exhibited synergistic cytotoxic effects in HepG2 and SMMC7721 cells. Meanwhile, L-arginine increased 5-FU inhibitory effect on HepG2 and SMMC7721 cells by increasing NO production. Co-treatment with L-arginine and 5-FU resulted in a significant decrease in both G6PDH and LDH enzymatic activities, as well as reduced levels of ATP and LD compared to treatment with L-arginine or 5-FU alone. Moreover, the combination of L-arginine and 5-FU resulted in a decrease in the expression of GLUT1, PKM2, LDHA, p-PI3K and p-AKT. Furthermore, the combination demonstrated a synergistic effect in downregulating the expression of HIF-1α and β-catenin, which were further diminished upon the addition of shikonin, a specific inhibitor of PKM2. LY294002 treatment further reduced the expression of GLUT1, PKM2, and LDHA proteins induced by combined L-arginine and 5-FU treatment compared to the combined group. However, the reduction in p-PI3K, p-AKT, and GLUT1 expression caused by L-arginine and 5-FU combination was also reversed in HepG2 and SMMC7721 cells with iNOS knockdown, respectively. Additionally, the combination of L-arginine and 5-FU led to a greater reduction in the enzymatic activity of ALT, AST, G6PDH and LDH, as well as a significant reduction in hepatic index, AFP, AFP-L3, ATP and LD levels in a rat model of HCC. Moreover, the simultaneous administration of L-arginine and 5-FU significantly improved the gross morphology of the liver, reduced nuclear atypia, inhibited the proliferation of cancer cells, and decreased the expression levels of p-PI3K, p-AKT, GLUT1, PKM2, and LDHA, while iNOS expression was increased in the combination group. Taking together, L-arginine and 5-FU combination resulted in the inhibition of enzymes in aerobic glycolysis via the iNOS/NO/AKT pathway, which led to the suppression of glucose metabolism and downregulation of nuclear transcription factors, thereby impeding the proliferation of hepatocellular carcinoma cells.

## 1 Introduction

According to the latest global cancer statistics (2020), hepatocellular carcinoma (HCC) is the fifth most common cancer and the second leading cause of cancer-related mortality among men globally while in most high-risk HCC areas such as China, South Korea, and sub-Saharan Africa, chronic HBV infection, aflatoxin exposure, or both are the primary risk factors (Sung et al., 2021). Bevacizumab and atezolizumab combination have become the first-line treatment choice for HCC patients who have not previously received systemic therapy. It is used in cases of unresectable hepatocellular carcinoma (Finn et al., 2020). As a multi-targeted tyrosine kinase inhibitor, sorafenib has been approved for indications in renal cell carcinoma, hepatocellular carcinoma, thyroid cancer, and more. It is used as a standard first-line treatment for unresectable HCC(Kim et al., 2017). However, FOLFOX4 (fluorouracil, calcium folinate, oxaliplatin) may still be considered the optimal treatment choice in many developing countries due to its effectiveness (Oranratnachai et al., 2021). In China, the FOLFOX4 regimen is approved for first-line treatment of locally advanced and metastatic HCC in patients who are not suitable for surgical resection or local treatment (Qin et al., 2014). Therefore, 5-fluorouracil (5-FU) remains one of the main drugs in HCC chemotherapy regimens.

Recent studies revealed that the aerobic glycolysis, as an additional hallmark of cancer, may play a role in the hepatocarcinogenesis (Schwartz et al., 2017; Jiang et al., 2020). Aerobic glycolysis, also termed the Warburg effect, first described by Otto Heinrich Warburg in 1924. Unlike normal cells that produce energy through a comparatively low rate of glycolysis followed by oxidation of pyruvate in the mitochondria, cancer cells predominantly produce energy through a high rate of glycolysis followed by lactic acid fermentation in the cytosol even when oxygen is sufficient. Despite being less efficient in terms of ATP production, this metabolic reprogramming provides cancer cells with a growth advantage by facilitating the production of biosynthetic precursors and maintaining redox balance. In aerobic glycolysis, cells exhibit low efficiency in utilizing glucose, necessitating higher glucose intake. Upregulation of glycolysis has been observed in many primary and metastatic cancers, supporting increased glucose consumption in cancer cells (Li and Zhang, 2016). Numerous studies have confirmed that even in the presence of oxygen, cancer cells exhibit enhanced aerobic glycolysis (Lunt and Vander Heiden, 2011). The upregulation of glycolysis leads to acidosis in the tumor microenvironment, which increases the demand for phenotypic adaptation toward acid-induced toxicity (Honigova et al., 2022). These resistant cell populations gain a significant growth advantage by adapting to the acidic environment. Conversely, due to natural cell plasticity in this environment, acidosis enhances the invasiveness of cancer, thus providing a growth advantage to cancer cells due to their metabolic shift toward glycolysis. To compensate for the lower energy production resulting from aerobic glycolysis compared to oxidative phosphorylation, cancer cells enhance glucose uptake by upregulating glucose transporters, notably glucose transporter 1 (GLUT1), as well as several key glycolytic enzymes, including pyruvate kinase M2 (PKM2) and lactate dehydrogenase A (LDHA) (Feng et al., 2020; Chen et al., 2022).

Glucose transporter proteins (such as GLUTs) are believed to be overexpressed to promote glucose consumption, especially in advanced and metastatic stages. GLUT1 has a high affinity for glucose and is significantly overexpressed in cancers such as brain cancer, breast cancer, cervical cancer, colorectal cancer, and bladder cancer (Abdel-Wahab et al., 2019). PKM2 is overexpressed in various cancer types and regulates the redox homeostasis in cancer cells. LDH is a key enzyme that converts pyruvate to lactate, and the accumulation of lactate significantly lowers intracellular pH. Lactic acid plays a crucial role in both aerobic and anaerobic conditions in cancer and metastasis (Paul et al., 2022). Serum lactate dehydrogenase serves as a prognostic factor for breast cancer and non-small cell lung cancer (Gong et al., 2019; Liu et al., 2019). Research indicates that LDHA is overexpressed in several tumor cells, and its inhibitory effects can significantly slow down tumor progression (Alobaidi et al., 2023).

The PI3K-AKT pathway is a cellular signaling pathway that regulates various cellular processes such as cell growth, proliferation, and survival. It is activated by various growth factors and hormones and plays a key role in regulating metabolism. Activation of the AKT pathway has been demonstrated to promote aerobic glycolysis (Courtnay et al., 2015; Pi et al., 2022). Furthermore, activation of the PI3K/AKT signaling pathway can induce the expression of glucose transporters GLUT1 and GLUT4, leading to an increased rate of glucose uptake in cancer cells (Melstrom et al., 2008; Park et al., 2020). Therefore, Inhibition of the AKT pathway to impede the process of aerobic glycolysis may provide significant therapeutic benefits in the treatment of hepatocellular carcinoma (HCC).

5-Fluorouracil (5-FU) is an antimetabolite that interferes with DNA synthesis and cell proliferation by misincorporating fluoronucleotides into DNA and RNA, and by inhibiting the thymidylate synthase (TS), thereby disrupting the normal functioning of cancer cells and prevent their growth and division (Longley et al., 2003). Some evidence revealed that glucose metabolism may play a role in the effectiveness of 5-FU treatment: glycolysis inhibition sensitizes lung cancer cells to 5-FU; targeting the Warburg effect in KRAS G12D glycolytic tumor organoids enhances 5-FU toxicity by further altering the nucleotide pool (Zhao et al., 2014; Ludikhuize et al., 2022). Thus, inhibiting glycolysis may be a promising approach to increase the effectiveness of 5-FU in cancer treatment.

Tumor cell proliferation is a critical step in tumorigenesis. L-arginine is a crucial immunomodulatory amino acid involved in both innate and adaptive immune responses. It participates in T cell activation by upregulating T cell receptors and accelerating cell cycle progression. Supplementing arginine can shift T cell metabolism from glycolysis to oxidative phosphorylation (OXPHOS), promoting T cell survival and enhancing its anti-tumor capabilities (Chen et al., 2021). L-arginine has been shown to exert inhibitory effects on tumor cell proliferation in various cancers, including breast cancer, mastocytoma, neuroblastoma, squamous cell carcinoma, pheochromocytoma, colon cancer, and pancreatic cancer (Ma et al., 2010). Two key enzymes involved in L-arginine metabolism, inducible nitric oxide synthase (iNOS) and arginase, compete for L-arginine to regulate innate immune responses. L-arginine as a precursor of nitric oxide (NO), is converted to NO and L-citrulline in the presence of inducible nitric oxide synthase (iNOS). High concentration of NO would show a higher anti-cancer effect through influence the DNA damage repair response, and modulate cell cycle arrest (Khan et al., 2020). Arginase, a urea cycle enzyme, converts L-arginine into ornithine and urea, thereby limiting its availability for NOS (Caldwell et al., 2015). In MCF7 breast cancer cells, the inhibitory effect of statins is mediated by suppressing arginase activity, reducing the utilization of L-arginine as a polyamine precursor, and simultaneously inducing nitric oxide (NO) production via inducible nitric oxide synthase (iNOS) (Kotamraju et al., 2007).The statins may reduce the risk of HCC is related to increased NADPH formation, which serves as a coenzyme for NOS, leading to enhanced NO production (Erbas et al., 2015). Consequently, enhancing iNOS activity to generate high levels of NO has become a potential direction for antitumor therapy. 5-FU significantly increased the iNOS expression to 0.1687 +/− 0.01968 (*p* < 0.05, vs. control group), and the concentration of nitric oxide to 213 +/− 30.2 mmol/L (*p* < 0.05, vs. control group) in human liver carcinoma Bel7402 cell line (Jiang et al., 2002). Endogenously produced nitric oxide plays a critical role in inducing apoptosis in hepatocellular carcinoma cells through the use of 5-FU. L-arginine may serve as a valuable adjuvant to enhance the efficacy of 5-FU chemotherapy by increasing nitric oxide production.

In the present study, we elucidated the underlying mechanisms of L-arginine (a NO precursor) in combination with 5-FU through changing the process of aerobic glycolysis. The results demonstrated that 5-FU increased the expression of iNOS protein in HepG2 and SMMC7721 cells, allowing L-arginine to produce high concentrations of NO, thereby inhibiting the process of aerobic glycolysis through the PI3K/AKT pathway.

## 2 Results

### 2.1 Effect of L-arginine and 5-fluorouracil combination on proliferation of HCC cells

**FIGURE 1:**
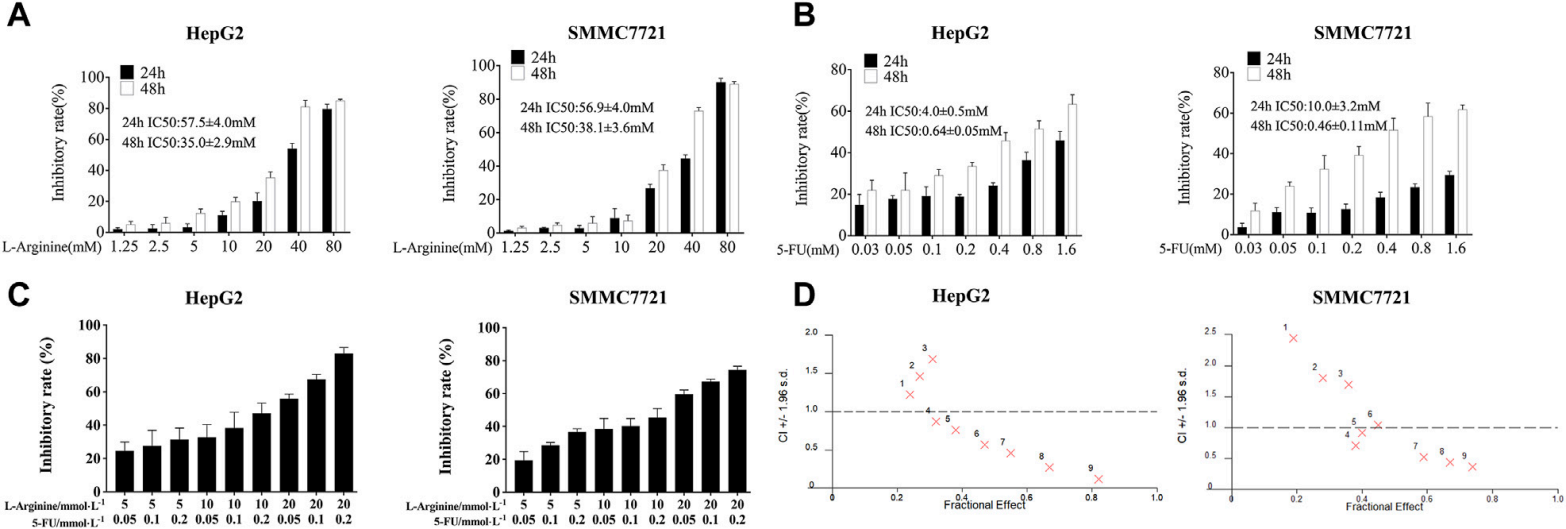
Effect of L-arginine and/or 5-fluorouracil on the proliferation of HepG2 and SMMC7721 cells. **(A)** Effect of L-arginine on cell inhibition for 24 h and 48 h through MTT assay. **(B)** Effect of 5-fluorouracil on cell inhibition for 24 h and 48 h through MTT assay. **(C)** Effect of L-arginine and 5-fluorouracil combination on cell inhibition for 48 h through MTT assay. **(** **D** **)** The combination index (CI) of the combined medication was calculated using CalcuSyn software. Data were mean ± SD. *n* = 3 for each concentration.

After 24 and 48 h of treatment with different concentrations of L-arginine (1.25, 2.5, 5, 10, 20, 40, 80 mM) and 5-FU (0.03, 0.05, 0.1, 0.2, 0.4, 0.8, 1.6 mM) on HepG2 and SMMC7721 cells, the proliferation ability of HCC cells was inhibited in a concentration and time-dependent manner (Figures 1A,B). Furthermore, 5-FU showed a significant inhibitory effect at the 48-h treatment time. As a result, a drug treatment duration of 48 h was selected for subsequent combination experiments. L-arginine (5, 10, 20 mM) and 5-FU (0.05, 0.1, 0.2 mM) were selected as the combined concentrations of L-arginine and 5-FU according to their respective half-inhibitory concentrations (IC50), which significantly increased the inhibitory rate in HepG2 and SMMC7721 cells (Figure 1C). To verify the synergistic anticancer effect of L-arginine (5, 10, 20 mM) and 5-FU (0.05, 0.1, 0.2 mM), CI values were calculated. Co-treatment with L-arginine (20 mM) and 5-FU (0.2 mM) resulted in significant inhibition of proliferation in both cell lines, with the lowest CI value observed (CI values < 0.4) (Figure 1D; Table 1; Table 2). Consequently, these concentrations were selected for further combination studies. Taken together, these finding indicate that L-arginine plus 5-FU exerted synergistic cytotoxic effects on HepG2 and SMMC7721 cells.

**TABLE 1:** Combination index of the combined medication in HepG2 cells.

	L-arginine (mM)	5-FU (mM)	Fa	CI
1	5	0.05	0.24	1.23
2	5	0.1	0.27	1.46
3	5	0.2	0.31	1.69
4	10	0.05	0.32	0.87
5	10	0.10	0.38	0.76
6	10	0.20	0.47	0.57
7	20	0.05	0.55	0.46
8	20	0.10	0.67	0.27
9	20	0.20	0.82	0.12

CI, combination index; Fa, fraction affected.

**TABLE 2:** Combination index of the combined medication in SMMC7721 cells.

	L-arginine (mM)	5-FU (mM)	Fa	CI
1	5	0.05	0.19	2.44
2	5	0.10	0.28	1.81
3	5	0.20	0.36	1.70
4	10	0.05	0.38	0.72
5	10	0.10	0.4	0.91
6	10	0.20	0.45	1.05
7	20	0.05	0.59	0.53
8	20	0.10	0.67	0.44
9	20	0.20	0.74	0.37

CI, combination index; Fa, fraction affected.

### 2.2 Effects of L-arginine and 5-fluorouracil combination on NO level and iNOS expression in HCC cells

**FIGURE 2:**
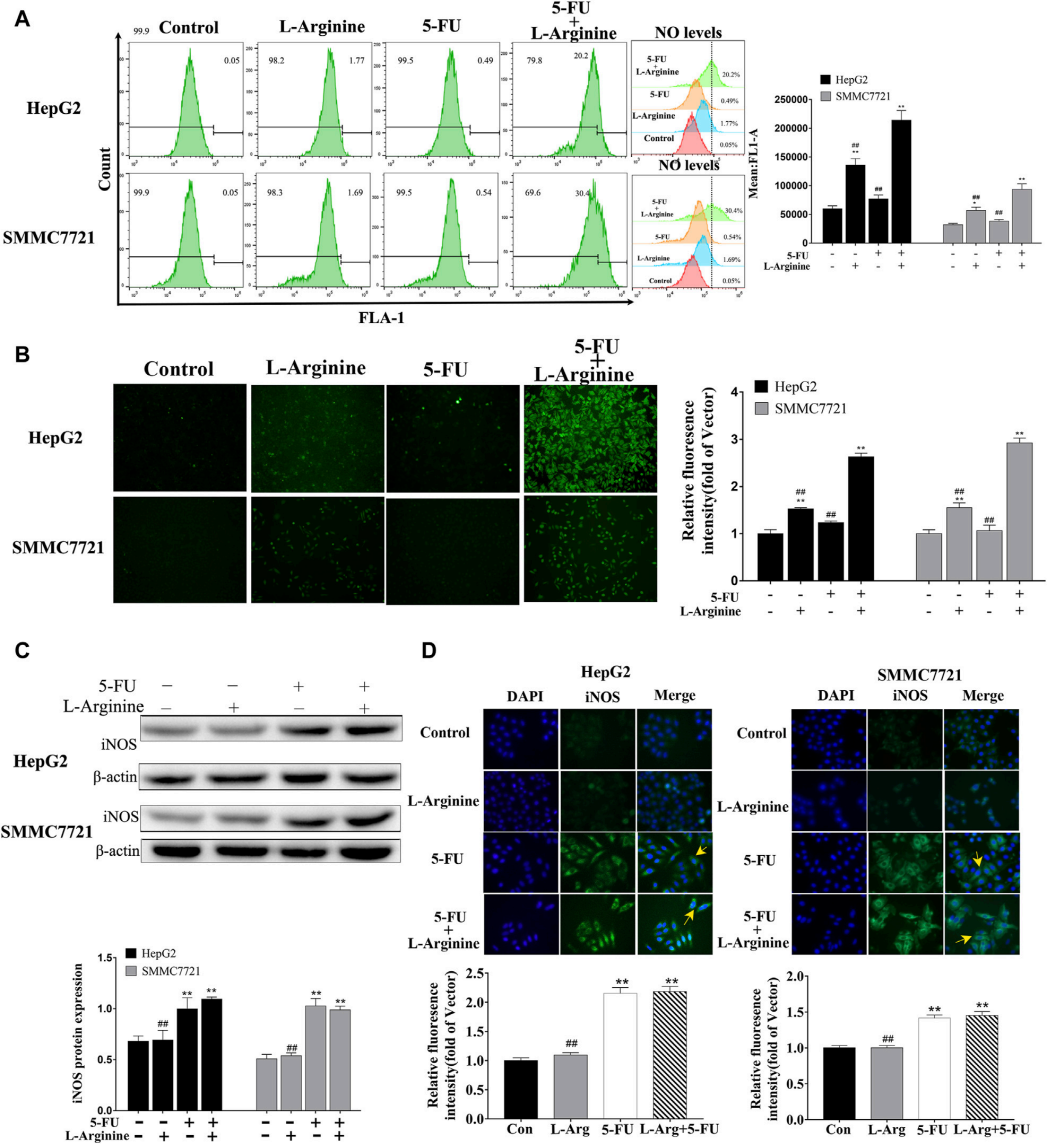
Effect of L-arginine and 5-fluorouracil combination on NO level and iNOS expression in HepG2 and SMMC7721 cells. **(A,B)** Effect of L-arginine and 5-fluorouracil combination on NO level through both flow cytometry and fluorescence microscope. **(C,D)** Effect of L-arginine and 5-fluorouracil combination on iNOS expression using Western blot and immunofluorescence assay. Data were mean ± SD. *n* = 3 for each concentration. *p < 0.05, **p < 0.01, vs. control group. ^**#**^ *p* < 0.05, ^**##**^ *p* < 0.01, vs. L-arginine and 5-FU combination group.

To investigate whether the synergistic anticancer effects of L-arginine plus 5-FU were related to NO level, DAF-FM DA as a NO fluorescent probe was used to label NO in HepG2 and SMMC7721 cells. Flow cytometry analysis of NO fluorescence also revealed an increase in NO fluorescence in the combination group, with a rightward shift of the fluorescence peak (Figure 2A). As shown in Figures 2A,B significant increase in NO levels was observed in the group treated with the combination of L-arginine and 5-fluorouracil under fluorescence microscopy. Additionally, both Western blot and immunofluorescence analysis showed a significant increase in iNOS expression in both the 5-FU group and the group treated with the combination of L-arginine and 5-FU compared to the control group (Figures 2C,D). These findings indicate that the elevation of NO levels following combined treatment with L-arginine and 5-FU may be associated with the upregulation of iNOS expression by 5-FU.

### 2.3 Effects of L-arginine and 5-fluorouracil combination on glucose metabolism in HCC cells

**FIGURE 3:**
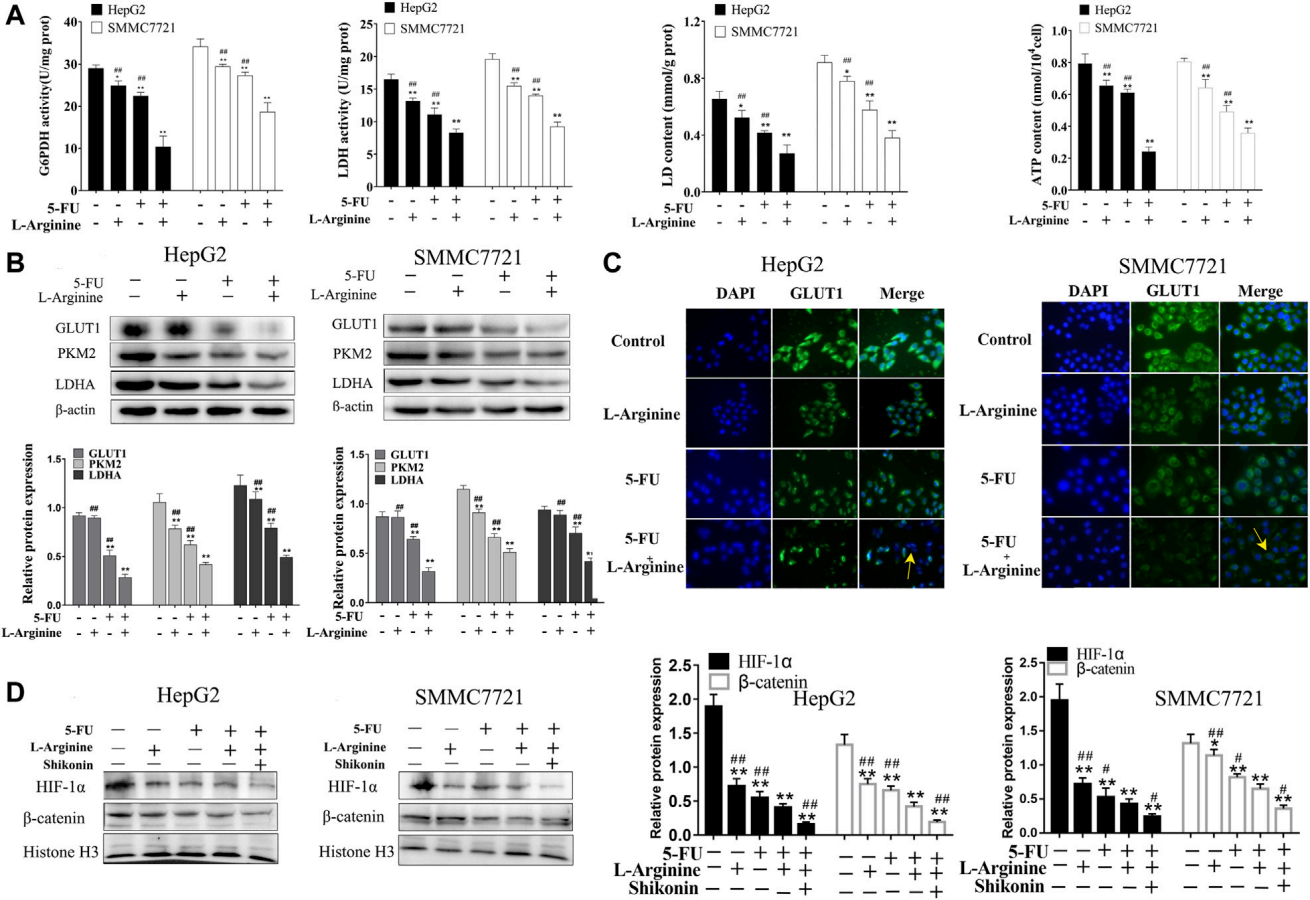
Effects of L-arginine and 5-fluorouracil combination on glucose metabolism and nuclear transcription factor in HepG2 and SMMC7721 cells. **(A)** Effect of L-arginine and 5-fluorouracil combination on activity of G6PDH and LDH, levels of LD and ATP. **(B)** Effect of L-arginine and 5-fluorouracil combination on the expressions of GLUT1, PKM2, and LDHA using Western blot. **(C)** Effect of L-arginine and 5-fluorouracil combination on the expression of GLUT1 using immunofluorescence assay. **(D)** Effect of PKM2 inhibitor on the expressions of HIF-1α and β-catenin using Western blot. Cells were pre-treated with a PKM2 inhibitor (shikonin, 2 μM) for 1 h prior to the addition of L-arginine and 5-fluorouracil. Data were mean ± SD. *n* = 3 for each concentration. *p < 0.05, **p < 0.01, vs. control group. ^#^ *p* < 0.05, ^##^ *p* < 0.01, vs. L-arginine and 5-FU combination group.

Colorimetric assays were used to measure the enzymatic activity of G6PDH and LDH, as well as the levels of LD and ATP in HepG2 and SMMC7721 cells. Compared to treatment with L-arginine or 5-FU alone, co-treatment with L-arginine and 5-FU resulted in a significant decrease in both G6PDH and LDH enzymatic activities, as well as reduced levels of ATP and LD (Figure 3A). Immunofluorescence and Western blot analysis were used to determine the expression of proteins related to glucose metabolism in HepG2 and SMMC7721 cells. Compared to treatment with L-arginine or 5-FU alone, co-treatment with L-arginine and 5-FU resulted in a significant decrease in GLUT1 protein expression, as determined by both immunofluorescence and Western blot analysis (Figures 3B,C). Additionally, expression of other proteins, such as PKM2 and LDHA, was also significantly reduced in the group treated with the combination of L-arginine and 5-FU. Furthermore, co-treatment with L-arginine and 5-FU could reduce the expression of HIF-1α and β-catenin proteins compared with L-arginine or 5-FU group (Figure 3D). After adding the shikonin (a PKM2-specific inhibitor), the expressions of HIF-1α and β-catenin proteins were further reduced compared with combined group. These results suggested that the concurrent administration of the two drugs not only suppressed the glycolytic pathway, leading to a decrease in PKM2 expression, but also modulated nuclear transcription factors such as HIF-1α and β-catenin, thereby reducing their protein expression and ultimately curtailing cell proliferation.

### 2.4 Effects of L-arginine and 5-fluorouracil combination on PI3K/AKT pathway in HCC cells

**FIGURE 4:**
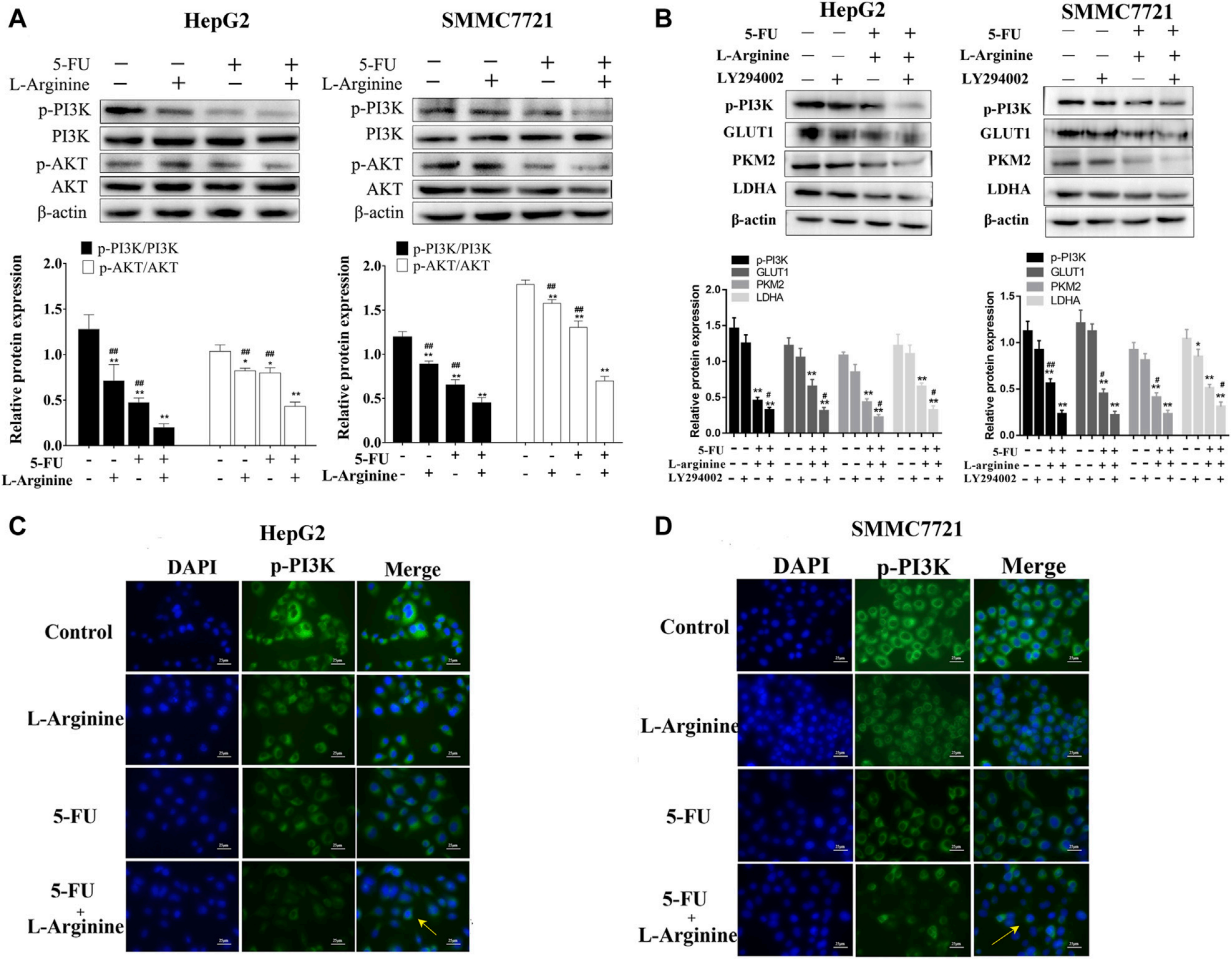
Effect of L-arginine and 5-fluorouracil combination on the expressions of PI3K/AKT pathway. **(A)** Effect of L-arginine and 5-fluorouracil combination on the expressions of p-PI3K and p-AKT using Western blot. **(B)** Effect of PI3K inhibitor on the expressions of p-PI3K, GLUT1, PKM2, and LDHA using Western blot. Cells were pre-treated with LY294002 (10 μM) for 1 h prior to the addition of L-arginine and 5-fluorouracil. **(C,D)** Effect of L-arginine and 5-fluorouracil combination on the expression of p-PI3K using immunofluorescence assay in HepG2 and SMMC7721 cells. Data were mean ± SD. *n* = 3 for each concentration. *p < 0.05, **p < 0.01, vs. control group. ^#^ *p* < 0.05, ^##^ *p* < 0.01, vs. L-arginine and 5-FU combination group.

As shown in Figure 4, L-arginine plus 5-FU led to a significant decrease in the expression of both p-PI3K and p-AKT proteins compared with either drug alone, as revealed by Western blot analysis and immunofluorescence assays. LY294002 treatment further reduced the expression of GLUT1, PKM2, and LDHA proteins induced by combined L-arginine and 5-FU treatment compared to the combined group. These results suggested a potential relationship between the combined treatment of L-arginine and 5-FU and the potentiation of the inhibitory effect on glucose metabolism in HepG2 and SMMC7721 cells via the PI3K/AKT pathway.

### 2.5 Effect of iNOS on glucose metabolism proteins and of PI3K/AKT pathway in HCC cells treated with L-arginine and 5-fluorouracil

**FIGURE 5:**
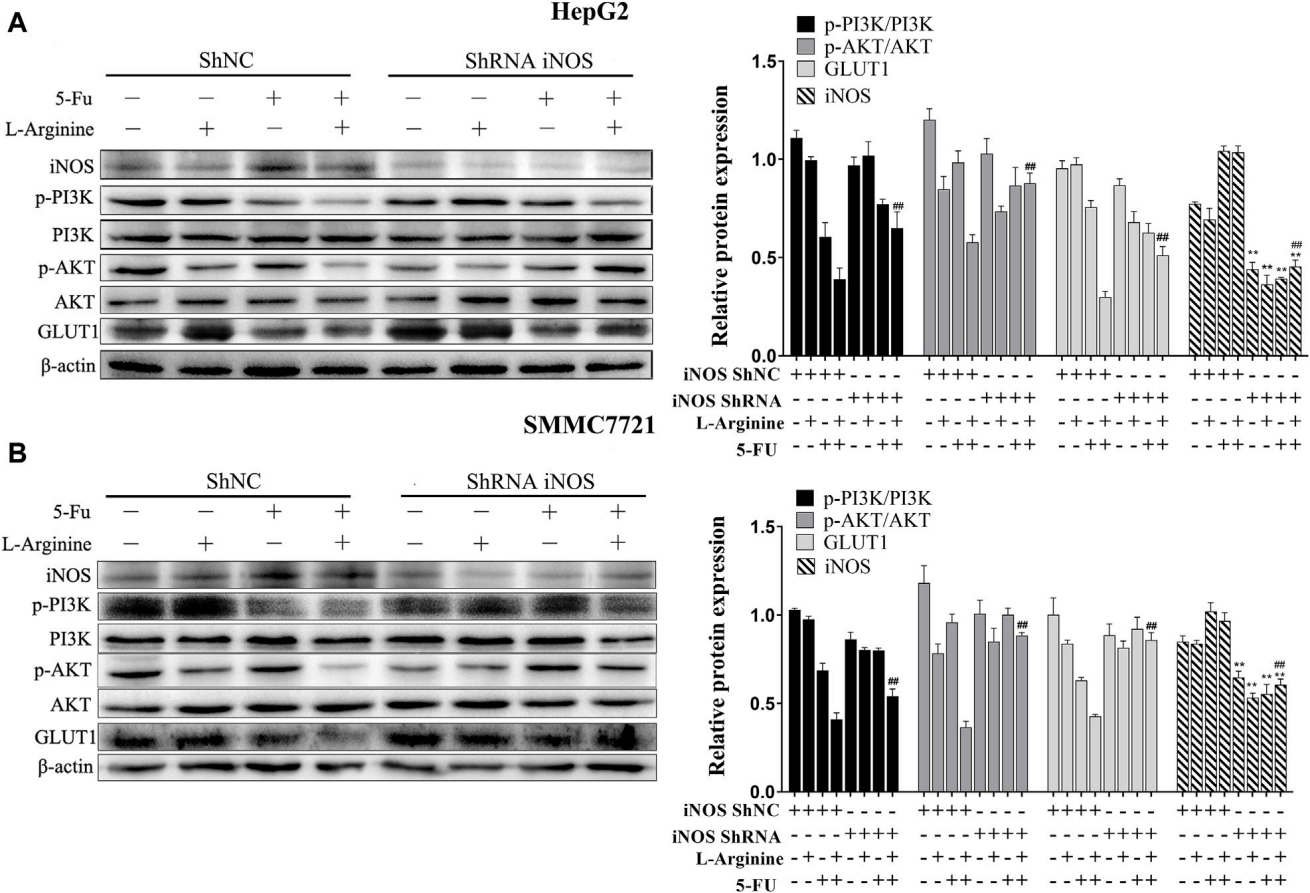
Effect of iNOS shRNA on glucose metabolism and PI3K/AKT pathway after treatment of L-arginine and 5-fluorouracil in HepG2 and SMMC7721 cells. **(A)** The effect of iNOS shRNA on the expressions of iNOS, p-PI3K, p-AKT and GLUT1 after treatment of L-arginine and 5-FU in HepG2 cells. **(B)** Effect of iNOS shRNA on the expressions of iNOS, p-PI3K, p-AKT and GLUT1 after treatment of L-arginine and 5-fluorouracil in SMMC7721 cells. Data were mean ± SD. *n* = 3 for each concentration. *p < 0.05, **p < 0.01 vs. control group of ShNC cells. ^**#**^ *p* < 0.05, ^**##**^ *p* < 0.01, vs. L-arginine and 5-FU combination group of ShNC cells.

To investigate the role of iNOS in mediating the synergistic effects of L-arginine and 5-fluorouracil on HepG2 and SMMC7721 cells, knockdown of iNOS through shRNA was introduced. In HepG2 and SMMC7721 cells with iNOS knockdown, the decrease in p-PI3K and p-AKT expression induced by the combined treatment of L-arginine and 5-FU was reversed. Similarly, the reduction in GLUT1 expression caused by the concomitant administration of L-arginine and 5-FU was also reversed in cells with iNOS knockdown (Figure 5). Therefore, these data indicated that the inhibitory effect of the combined treatment of L-arginine and 5-FU on the PI3K/AKT pathway and glucose metabolism was associated with high iNOS expression.

### 2.6 Effect of L-arginine and 5-fluorouracil combination on biochemical markers, morphological changes and proliferation in primary rat hepatocellular carcinoma

**FIGURE 6:**
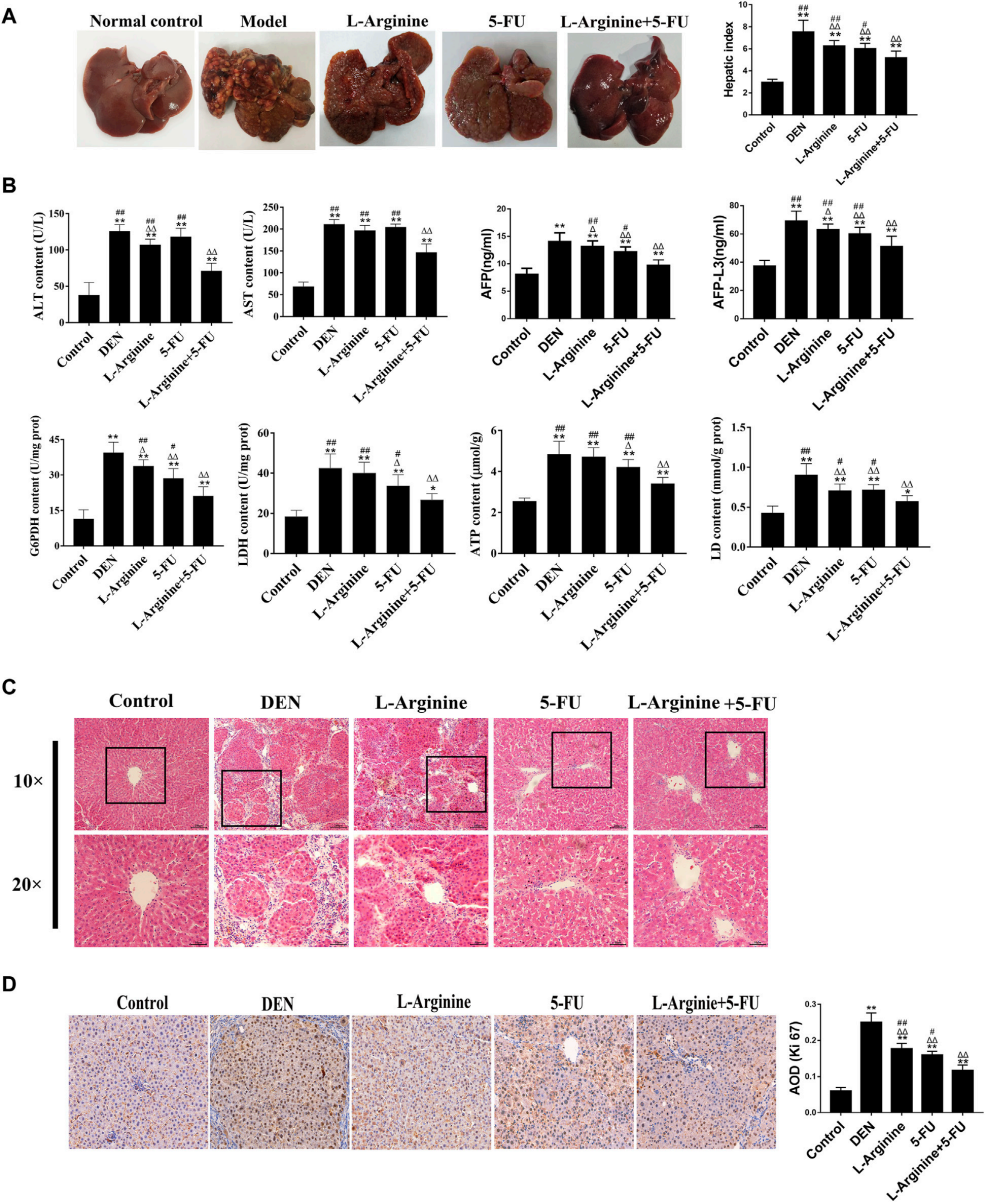
Effect of L-arginine and 5-fluorouracil combination on the biochemical markers, morphological changes, and proliferation in primary rat hepatocellular carcinoma. **(A)** Effect of L-arginine and 5-fluorouracil combination on the gross morphology and hepatic index of the rat liver. **(B)** Effect of L-arginine and 5-fluorouracil combination on the biochemical markers in primary rat hepatocellular carcinoma. **(C)** Effect of L-arginine and 5-fluorouracil combination on hepatic morphological changes using HE staining. **(D)** Effect of L-arginine and 5-fluorouracil combination on cell proliferation using IHC staining of Ki67. Data were mean ± SD, *n* = 12 for each concentration. *p < 0.05, **p < 0.01, vs. normal control group. ^**△**^ *p* < 0.05, ^**△**^ *p* < 0.01, vs. DEN model group. ^**#**^ *p* < 0.05, ^**##**^ *p* < 0.01, vs. L-arginine and 5-FU combination group.

A primary rat hepatocellular carcinoma induced by DEN was constructed to testify the anticancer effect of L-arginine and 5-FU combination. As shown in Figure 6A, the liver surface of the normal control group rats was smooth and the texture soft; the liver surface presented with nodules of varying sizes and greater number in the model group; in the L-arginine group, the 5-FU group, and the combined L-arginine and 5-FU group, the number of nodules on the liver surface was reduced, tumor growth decreased, and the color tended towards normal. The combination of L-arginine and 5-FU also significantly reduced the levels of hepatic index compared to L-arginine or 5-FU alone. As shown in Figure 6B, the combination of L-arginine and 5-FU can significantly reduce the activities of ALT and AST in serum compared to L-arginine or 5-FU alone. Furthermore, the combined treatment of L-arginine and 5-FU led to a greater reduction in AFP and AFP-L3 levels compared to treatment with either L-arginine or 5-FU alone. Additionally, the combined use of L-arginine and 5-FU led to a greater reduction in the enzymatic activity of G6PDH and LDH, as well as a significant reduction in ATP and LD levels compared to treatment with either L-arginine or 5-FU alone. HE staining was used to evaluate the effects of co-administration of L-arginine and 5-FU on the pathological morphology of liver tissue in the rat model of hepatocellular carcinoma. As shown in Figure 6C, the following morphological changes were observed in the DEN-treated group: disordered liver lobule structure, increased nuclear size, and nuclear atypia in the hepatic sinusoids after 16 weeks of DEN treatment. Additionally, fibrous tissue proliferation in the hepatic interstitium formed fibrous septa or pseudocapsules. In contrast, large areas of necrosis were observed and there was a reduction in nuclear atypia in the treatment with L-arginine and/or 5-FU group. Moreover, fibrous tissue in the hepatic interstitium was reduced or regressed and there was an increase in inflammatory cell infiltration. As shown in Figure 6D, L-arginine and 5-FU treatment caused the percentage of Ki-67 positive cells was significantly elevated compared to treatment with either L-arginine or 5-FU alone. These results suggested co-administration of L-arginine and 5-FU attenuated the severity of pathological changes and aggressive tumor growth.

### 2.7 Effect of L-arginine and 5-fluorouracil combination on PI3K/AKT and glucometabolic protein expression in primary rat hepatocellular carcinoma

**FIGURE 7:**
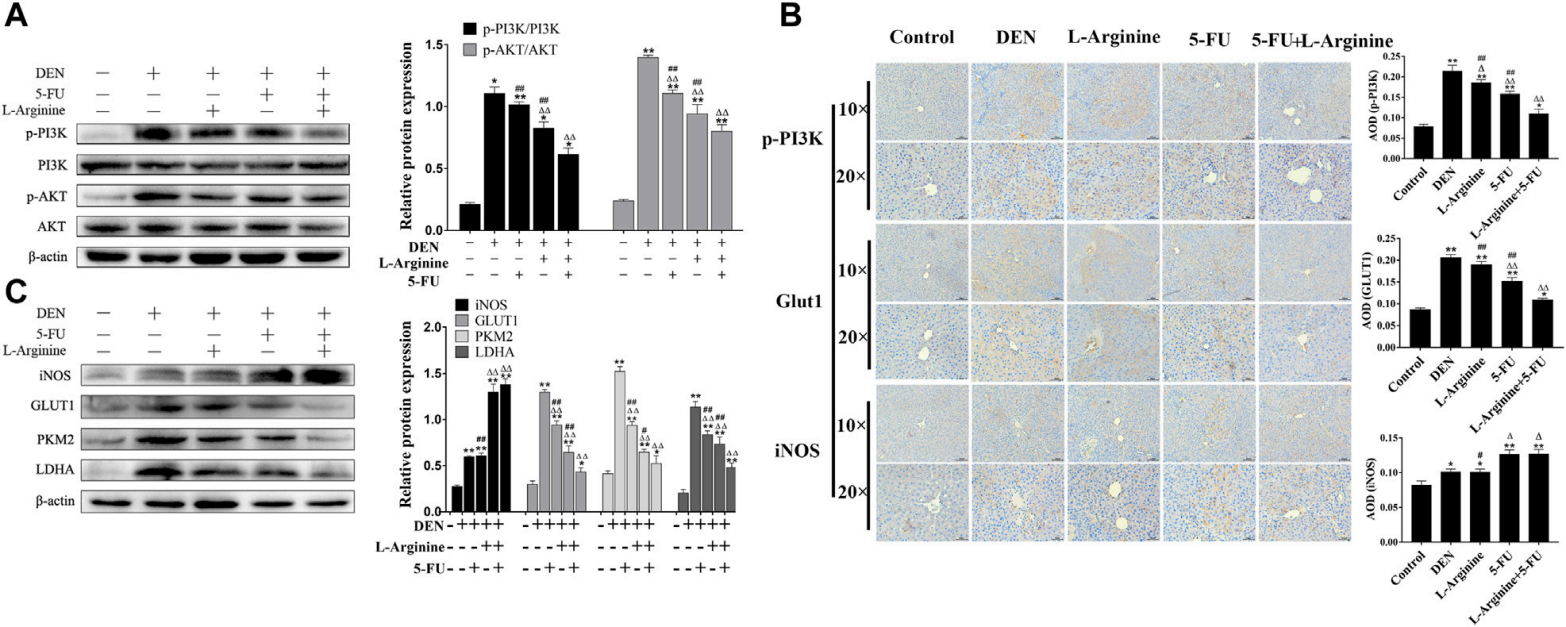
Effect of L-arginine and 5-fluorouracil combination on PI3K/AKT and glucometabolic protein expression in primary rat hepatocellular carcinoma. **(A)** Effect of L-arginine and 5-fluorouracil combination on expressions of p-PI3K and p-AKT using Western blot. **(B)** Effect of L-arginine and 5-fluorouracil combination on p-PI3K, GLUT1, and iNOS using immunohistochemical analysis. **(C)** Effect of L-arginine and 5-fluorouracil combination on expressions of iNOS, GLUT1, PKM2, and LDHA using Western blot. Data were mean ± SD, *n* = 12 for each concentration. *p < 0.05, **p < 0.01, vs. normal control group. ^**△**^ *p* < 0.05, ^**△**^ *p* < 0.01, vs. DEN model group. ^**#**^ *p* < 0.05, ^**##**^ *p* < 0.01, vs. L-arginine and 5-FU combination group.

As shown in Figure 7, Western blot analysis and immunohistochemical analysis revealed a significant increase in iNOS expression levels in the 5-FU and L-arginine combined with 5-FU groups compared to the DEN-treated group. In contrast, simultaneous administration of L-arginine and 5-FU resulted in a significant decrease in p-PI3K and p-AKT expression levels, as well as a significant reduction in the expression of GLUT1, PKM2, and LDHA compared to treatment with either L-arginine or 5-FU alone. Taking together, the simultaneous administration of L-arginine and 5-FU could inhibit PI3K/AKT pathway and glucose metabolism-related proteins in primary rat hepatocellular carcinoma.

## 3 Discussion

L-arginine plays a vital role in modulating immunity, impacting both the body’s natural and acquired defense mechanisms. Some studies revealed L-arginine enhances arginine deiminase induced human lymphoma cell growth inhibition through NF-kBp65 and p53 expression (Shu et al., 2014). In a solid Ehrlich carcinoma (SEC) mouse model, L-arginine increased survival rates, elevated total nitrate/nitrite levels in tissues, upregulated p53, JNK, beclin-1, and caspase-3 expression, and improved histopathological features (Kabel et al., 2021). The cytostatic effect of relaxin on MCF-7 breast cancer cells may rely on its ability to stimulate endogenous production of NO through activation of the L-arginine-NO pathway (Bani et al., 1995).In addition, L-Arginine induces NO production and potentiates the anticancer effect of 5-FU in Breast Adenocarcinoma (Bala and Panditharadyula, 2019). In this study, we aimed to assess the inhibitory effect of L-arginine and 5-FU on HCC cells. When L-arginine was at a concentration of 5 mM, and 5-FU concentrations were 0.05 mM, 0.1 mM, and 0.2 mM, the combination index (CI) values were all greater than 1. However, when L-arginine was increased to 10 mM or higher, the CI values become less than 1. These results suggested that the synergistic effect between 5-FU and increased intracellular iNOS expression occurs only when L-arginine reached a sufficient concentration, leading to a noticeable increase in NO levels. Therefore, concentrations of L-arginine (5, 10, 20 mM) and 5-FU (0.05, 0.1, 0.2 mM) were selected for combined treatment based on the IC50 values determined by MTT assay after 48 h of drug exposure. The combination index (CI) was calculated using CalcuSyn software and it was found that the combination of L-arginine (20 mM) and 5-FU (0.2 mM) had the lowest CI value, indicating the significant inhibitory effect on HCC cells when used in combination.

Nitric oxide is synthesized by nitric oxide synthase NOS enzymes through a series of redox reactions involving the L-arginine. NOS1 and NOS3 are constitutively expressed, and produce small quantity of NO, while NOS2 generates NO for extended periods of time, at high concentrations, being key as regulator and effector during inflammation and infection (Dios-Barbeito et al., 2022). NOS2, also known as inducible nitric oxide synthase (iNOS), is an enzyme that locally produces high concentrations of NO in a short time frame and does not require calcium. The present data indicated that a significant increase in NO fluorescence intensity was observed in the combined L-arginine and 5-FU treatment group. Additionally, the intracellular fluorescence intensity in the L-arginine group was observed to be higher than that of both the control and 5-FU groups. This may be attributed to the role of L-arginine as a substrate for cellular NO synthesis, with increased L-arginine uptake resulting in elevated NO production. Moreover, a significant increase in iNOS protein expression was observed in HCC cells treated with 5-FU or a combination of L-arginine and 5-FU, while no significant change was observed in the L-arginine group. This suggests that 5-FU can increase the expression of iNOS protein in HCC cells, which can lead to the production of high concentrations of NO from L-arginine. These observations were consistent with the results obtained from MTT assay.

Deviant metabolism, including aerobic glycolysis and augmented anabolic pathways, is a prominent feature of cancer and plays a crucial role in tumorigenesis, metastasis, drug resistance, and the maintenance of cancer cells (Park et al., 2020). Overexpression of oncogenes in tumor cells has been shown to activate glycolytic enzymes. Glucose enters cells through glucose transporters (GLUTs) and is subsequently phosphorylated by hexokinases (HKs) in the cytoplasm, trapping it within the cell. During this metabolic process, oncogenes such as YAP, KRAS, and c-MYC increase the expression of GLUT1 in cancer cells. After being converted into glucose-6-phosphate (G6P) by hexokinase (HK), glucose can either be metabolized through glycolysis or enter the pentose phosphate pathway (PPP). The PPP generates NADPH and precursors for lipid and nucleotide synthesis, providing tumor cells with the energy and substrates necessary for macromolecule production, thus promoting tumor growth (Tsouko et al., 2014). Glucose-6-phosphate dehydrogenase (G6PDH) catalyzes the first step in the PPP, while the inhibition of G6PDH prevents the glucose flux to PPP. In normal cells, the tumor suppressor protein p53 interacts with G6PDH to inhibit its activity. Conversely, G6PDH expression is elevated, indicative of an augmented flux through the PPP in neoplastic cells (Cho et al., 2018; Paul et al., 2022). The present study indicated L-arginine combined with 5-FU significantly inhibited the expression of GLUT1 and the activity of G6PDH in HepG2 and SMMC7721 cells, resulting in a decrease in the transport of glucose into cells and a reduction in the g conversion of glucose 6-phosphate to PPP. One of rate-limiting enzymes is pyruvate kinase (PKs) in the glycolytic pathway can catalyze the conversion of phosphoenolpyruvate (PEP) to ATP and pyruvate. In contrast to other isoforms of PKs, PKM2 is highly expressed in cancer cells and its upregulation has been related to a poorer prognosis (Shang et al., 2016). The activity of PKM2 is primarily regulated by its oligomeric state. PKM2 can exist as a tetramer, which has high catalytic activity and is located in the cytoplasm, where it rapidly converts PEP to pyruvate, increasing glycolytic flux and ATP production (Anastasiou et al., 2012). Alternatively, PKM2 can exist as a monomer or dimer with lower catalytic activity, which can translocate to the nucleus and act as a co-activator for several transcription factors, including NF-κB, β-catenin/c-Myc, HIF-1α, and STAT3 (Azoitei et al., 2016; van Niekerk and Engelbrecht, 2018).

PKM2 modulates the synthesis process by generating either inactive dimeric forms or active tetrameric forms, ensuring tumor survival in hypoxic and nutrient-deprived environments. Additionally, it enhances antioxidant stress resistance in cancer cells (Alquraishi et al., 2019). As a nuclear transcription factor, PKM2 can bind to β-catenin to promote its transcription (Dayton et al., 2016). Both are recruited to the promoter region of cell cycle protein D1 (CCND1), causing dissociation of histone deacetylase 3 (HDAC3), acetylation of histone H3, and upregulation of CCND1 and oncogene transcription factor c-Myc, thereby promoting cell proliferation. Additionally, one of the main characteristics of most tumors is hypoxia, which is signaled through HIF1α. HIF1α can activate transcription factors Snail and Twist, which are involved in the regulation of E-cadherin, promoting cell invasion and chemotherapy resistance (Hasan et al., 2018). In this study, we found that the combined use of L-arginine and 5-FU reduced the expression of PKM2, inhibiting the expressions of HIF-1α and β-catenin. Furthermore, the expression of HIF-1α and β-catenin proteins was further reduced after adding the Shikonin, suggesting that the combined use of L-arginine and 5-FU after inhibiting PKM2 expression could further regulate nuclear transcription factors, reducing their protein expression and inhibiting cell proliferation.

Poor vascularization within the tumor microenvironment can result in chronic nutrient deprivation and decreased oxygen concentrations (Eberhard et al., 2000; Hanahan and Weinberg, 2011). In response to these environmental stresses, cancer cells alter their metabolic pathways to efficiently capture external metabolites and optimize metabolic enzyme activity, thereby enhancing their ability to survive and adapt (Pavlova and Thompson, 2016; Park et al., 2020). Angiogenesis, the process of new blood vessel formation, is intricately linked to VEGF, a signaling protein that plays a pivotal role in angiogenesis, especially under hypoxic conditions. HIF can activate VEGF, thereby influencing angiogenesis. The expression of VEGF, dependent on HIF-1α, promotes the proliferation and migration of endothelial cells and vascular smooth muscle cells, leading to the induction of angiogenesis. In cancer-associated fibroblasts (CAFs), HIF-1α mediated extracellular matrix (ECM) remodeling and metabolic reprogramming support cell survival. These processes are crucial for tumor progression and angiogenesis under other pathological conditions. The sodium nitroprusside (SNP), a NO donor, downregulates the activity and synthesis of the VEGF promoter in vascular smooth muscle cells (VSMCs) by interfering with the binding of the AP-1 transcription factor (Kimura and Esumi, 2003). Other reports indicate that NO suppresses the hypoxic induction of the VEGF gene by eliminating the accumulation of HIF-1α protein in VSMCs and tumor cell lines, through weakening the binding activity of HIF-1α (Huang et al., 1999). In this study, the combined use of L-arginine and 5-FU reduced the expression of PKM2, leading to a decrease in HIF-1α expression. This result suggested that an increase in intracellular NO levels can reduce the accumulation of HIF-1α protein in tumor cells, potentially further inhibiting the VEGF-induced angiogenesis.

LDH converts pyruvate to lactate, rather than acetyl-CoA that is used as TCA cycle intermediate. Both LDHA and LDHB, two isoforms of LDH, are capable of catalyzing the conversion of pyruvate to lactate as well as the reverse reaction. The LDHA isoform is highly expressed due to its preference for catalyzing the conversion of pyruvate to lactate in many cancer cell lines (Valvona et al., 2016). In hypoxic tumor microenvironments, HIF-1α can induce the activation of LDHA (Cui et al., 2017). Our study demonstrated that the combination of L-arginine and 5-FU exerted a synergistic inhibitory effect on the glucose metabolism, including the suppression of the expression of PKM2 and LDHA, resulting in a decrease of lactate and ATP. It is well known that lactate, a byproduct of glycolysis, promotes tumor growth and metastasis (Paul et al., 2022). Although aerobic glycolysis appears to be less efficient in ATP production, the rapid glucose fermentation by glycolysis causes cancer cells to take up more glucose than normal cells (Yang et al., 2016; Ganapathy-Kanniappan, 2017). This study demonstrated that the combination of L-arginine and 5-FU reduced glucose transport into cells by decreasing GLUT1 expression, and inhibited the activity of key enzymes including G6PDH, PKM2, and LDHA, thereby reducing lactate production and ATP generation, ultimately suppressing aerobic glycolysis in HepG2 and SMMC7721 cells. Additionally, L-arginine combined with 5-FU suppressed the expression of G6PDH to affect PPP flux, which might decrease the generation of NADPH and enhance DNA damage in cancer cells.

Activation of the PI3K-AKT pathway in cancer cells, due to oncogenic mutations, alters cellular metabolism by enhancing the function of nutrient transporters and metabolic enzymes. This facilitates the anabolic needs of rapidly proliferating cells. AKT promotes glucose uptake through both GLUT1 and GLUT4, promotes hexokinase 2 (HK2) activity, at least in part, by increasing its association with a voltage-dependent anion channel at the outer mitochondrial membrane (Gottlob et al., 2001; Hoxhaj and Manning, 2020). AKT inhibitor LY294002 could suppress the expression of p-AKT, c-Myc, HK2, PKM2, and pro-caspase3 and inhibited the viability in HepG2 cells, suggesting the important role of AKT suppression in regulation of cancer metabolism (Shin et al., 2021). In this study, L-arginine combined with 5-FU restrained the expression of p-PI3K and p-AKT in HepG2 and SMMC7721 cells. When the expression of iNOS was downregulated, the combination of L-arginine and 5-FU attenuated the inhibitory effect on the expression of p-PI3K, p-AKT, and GLUT1 proteins in HepG2 and SMMC7721 cells, suggesting that the inhibitory effect of the combination on glucose metabolism in these cells is related to iNOS expression. Our *in vivo* studies demonstrated that the combination of L-arginine and 5-FU not only increased the expression of inducible iNOS but also inhibited aerobic glycolysis. This inhibition was achieved by downregulating the expression of GLUT1, PKM2, and LDHA, which led to reduced levels of ATP and lactate. These effects were associated with the suppression of the PI3K/AKT signaling pathway. L-arginine, a semi-essential amino acid synthesized in the liver, may be present in lower concentrations in patients with liver cancer due to impaired liver function. When combined with 5-FU chemotherapy, the addition of L-arginine can improve the endogenous production of NO in liver cancer cells, enhancing the therapeutic effect and increasing the patient’s anti-tumor capability. L-Arginine may serve as an effective adjuvant to 5-FU. The combination therapy of L-arginine and 5-fluorouracil can selectively act on cells. Research by Mozhgan et al. on the effect of both on breast cancer cells found that the combination of L-arginine and 5-FU increased cell survival rates in HUVEC but induced cell death in MDA-MB-468 cells; NO measurements showed an increase in this molecule in both cell lines; an assessment of metabolic changes and molecular docking indicated that glycolytic activity in cancer cells was reduced, but not in normal cells; a reduction in embryotoxicity was observed in the combined treatment (Jahani et al., 2019). These findings clearly demonstrated that L-Arginine inhibited cell death induced by 5-FU in HUVEC while not in breast cancer cells. Therefore, L-Arginine may help enhance the efficacy of 5-FU, allowing the drug to reach and act on cancer cells more effectively and mitigate the adverse reactions of 5-FU.

## 4 Materials and methods

### 4.1 Reagents

5-fluorouracil (5-FU) was purchased from Merck Drugs & Biotechnology (Darmstadt, Germany). Fluorouracil injection was available from Shanghai Xudong Haipu Pharmaceutical Co. Ltd (Shanghai, China). L-arginine hydrochloride injection was from Shanghai Xinyi Jinzhu Pharmaceutical Co., LTD (Shanghai, China). Diethylnitrosamine (DEN) was purchased from Shanghai Yien Chemical Technology Company (Shanghai, China). AFP ELISA Kit (rat) was from Enzyme-linked Biotechnology Company (Shanghai, China), and AFP-L3 ELISA Kit (rat) was obtained from ZCIBIO Technology company (Shanghai, China). L-arginine, diamidino-phenyl-indole (DAPI), glucose-6-phosphate dehydrogenase (G6PDH) activity assay kit, lacate dehydrogenase (LDH) activity assay kit, alanine transaminase (ALT) activity kit, and aspartate aminotransferase (AST) kit were obtained from Solarbio Science Technology Company. (Beijing, China). ATP content assay kit was purchased from Lanjeco Biotechnology Company (Beijing, China). LY294002 and 3-Amino, 4-aminomethyl-2′,7′-difluorescein, diacetate (DAF-FM DA) were originated from Beyotime Biotechnology Company (Shanghai, China). Control small interfering RNA and iNOS-shRNA were purchased from Shanghai Genechem Company. (Shanghai, China). Primary antibodies against p-PI3K, LDHA and GLUT1 were originated from Biosynthesis Biotechnology Company. (Beijing China). Antibodies against Ki67, iNOS and β-actin were purchased from Proteintech Company (Wuhan, China). PI3K and AKT were obtained from Santa Cruz Biotechnology (California, United States); phospho-AKT and PKM2 were from Wanlei biotechnology Company (Shenyang, China); HIF-1α and β-catenin were bought from Cell Signaling Technology (Boston, United States); Shikonin was purchased from Med Chem Express (NJ, United States). Alexa Fluor 488-conjugated affinipure goat anti-rabbit IgG (H + L) was originated from Proteintech Company (Wuhan, China); HRP-conjugated secondary antibodies, including goat anti-rabbit IgG and goat anti-mouse IgG, were from Cell Signaling Technology Company (MA, United States).

### 4.2 Cell culture

The human HepG2 and SMMC-7721 cell lines were obtained from Beyotime Biotechnology Company (Shanghai, China). The cells were cultured in DMEM medium containing 10% fetal bovine serum (FBS), 100 mg/mL streptomycin, and 100U/mL penicillin in a humidified incubator under 5% CO_2_ at 37°C.

### 4.3 MTT assay

HepG2 and SMMC7721 cells were seeded into 96-well cell plates at a final density of 1.0 × 10^4^ cells/well and incubated for 12 h. Various concentrations of L-arginine and/or 5-fluorouracil were then added to the wells and incubated for 24 h or 48 h. Afterward, 20 μL of MTT solution (5 mg/mL) was added to each well and incubated for 4 h. The medium containing MTT was then removed and 200 μL DMSO was added to dissolve the formazan crystals. The plate was shaken for 10 min in the dark and the absorbance was measured at 490 nm (A_490_). The cell inhibition rate (%) was calculated using the following formula: (1-Mean A_490__sample_/Mean A_490 control_) × 100%. The combination index (CI) of the combined medication was calculated using CalcuSyn software based on the cell inhibition rate. CI > 1 indicates antagonism, CI < 1 indicates synergy, and CI = 1 indicates an additive effect.

### 4.4 Nitric oxide assay

DAF-FM DA was utilized as a fluorescent indicator to measure intracellular NO levels. Cells were grown in 6-well plates until they reached 80% confluence. The cells were then washed with PBS and incubated with 5 μM DAF-FM DA at 37°C for 20 min. After incubation, the cells were washed with PBS and resuspended in PBS for the duration of the experiment. NO production was measured using and a fluorescence microscope and a flow cytometer with excitation and emission wavelengths of 495 nm and 515 nm, respectively.

### 4.5 Immunofluorescence assay

HepG2 and SMMC7721 cells were plated in 48-well plates at a density of 8 × 10^5^ cells/well and incubated for 12 h. The cells were then treated with L-arginine (20 mM) and/or 5-FU (0.2 mM) for 48 h. After treatment, the cells were fixed with 4% paraformaldehyde for 20 min and permeabilized with 0.5% Triton X-100 for 20 min. The cells were then blocked with 10% goat serum at room temperature for 30 min before being incubated with primary antibodies against iNOS (1:500), p-PI3K (1:500), and GLUT1 (1:200) at 4°C overnight. After incubation with the primary antibody, the cells were washed with PBS and incubated with Alexa Fluor 488-conjugated affinipure goat anti-rabbit IgG at a dilution of 1:100 for 1 h in the dark at room temperature. The nuclei were stained with DAPI for 5 min before the cells were visualized using a fluorescence microscopy.

### 4.6 Western blot analysis

After being pretreated with L-arginine (20 mM) and/or 5-FU (0.2 mM) for 48 h, the cells were harvested and lysed using an ice-cold cell lysis buffer from Beyotime Biotechnology (Shanghai, China). The proteins were mixed with loading buffer and heated at 95°C–100°C for 5–10 min to denature them. Equal amounts of protein were loaded onto a 12% SDS-PAGE gel and separated by electrophoresis for 2 h. The separated proteins were then transferred onto a PVDF membrane for 2 h. The membrane was blocked with 5% non-fat milk before being incubated with primary antibodies at 4°C overnight. After incubation with the primary antibodies, the membrane was incubated with HRP-conjugated goat anti-rabbit or goat anti-mouse IgG at 37°C for 1 h. The protein bands were visualized on the PVDF membrane using an ECL hypersensitive chemiluminescence solution and a Bio-Rad gel imaging and analysis system.

### 4.7 Cell transfection assay

The cells were seeded into 6-well plates at a density of 5 × 10^5^ cells/mL and incubated until they had attached and grown. The medium was then replaced with serum-free and antibiotic-free DMEM. Cells were transfected with either iNOS shRNA or a negative control shRNA using Lipofectamine 3000 transfection reagent (Thermo Fisher Scientific, Massachusetts, United Statesa) according to the manufacturer’s instructions.

The iNOS shRNA sequence was sense: CCA​GAA​GCA​GAA​TGT​GAC​CAT and the negative control shRNA sequence was sense: TTC​TCC​GAA​CGT​GTC​ACG​T. The transfection mixture was prepared by diluting the shRNA and transfection reagent in serum-free and antibiotic-free medium and incubating at room temperature for 20 min. The mixture was then added to the corresponding wells and the plates were incubated in a 5% CO_2_ incubator at 37°C for 8 h. Subsequently, the medium was replaced with fresh complete medium containing serum and the cells were incubated for an additional 24 h to allow for shRNA expression and gene silencing. Finally, the cells were treated with L-arginine (20 mM) and/or 5-FU (0.2 mM) for an additional 48 h.

### 4.8 Animals preparation

Sixty SD male rats (SD, 5–6 weeks old) that weighed between 180g and 220 g were obtained from Liaoning Changsheng Biotechnology Co., LTD. All rats were housed under standard animal housing conditions of 25°C ± 3°C, 55% ± 2% humidity, 12 h day/night cycle. This study was approved by the local ethics committee of Zhengzhou University for animal care and use. All the procedures performed and the care given to the rats complied with institutional guidelines.

The rats of normal control group were intraperitoneally injected with saline. In the experimental group, the rats were all intraperitoneally injected with DEN at 50 mg/kg twice a week for 4 weeks. From 5 to 16 weeks, the DEN injection changed to 50 mg/kg once a week. Among these DEN-treated groups, the rats were divided into the L-arginine group (intraperitoneally, 700 mg kg^−1^), the 5-fluorouracil group (intraperitoneally, 20 mg kg^−1^), and the L-arginine combined with 5-fluorouracilt group (intraperitoneally, L-arginine 700 mg·kg^−1^ and 5-fluorouracil 20 mg·kg^−1^). After 16 weeks, all rats were sacrificed and blood from each of the animals were collected. The hepatic index (HI) was calculated using the following formula: (Liver weight/Rat body weight) ×100%

### 4.9 Biochemical determinations

Serum was collected from the abdominal aorta of anesthetized rats and subsequently separated by centrifugation. The activities of ALT and AST were measured using respective kits according to the manufacturer’s instructions. After incubation for 10 min, the absorbance at 505 nm for both ALT and AST were determined spectrophotometrically.

Liver tissues were collected from all animals in the different experimental groups and stored at −80°C until further analysis. The activities of G6PDH and LDH, as well as the contents of ATP and LD in liver tissue, were analyzed using respective kits following the manufacturer’s protocols.

### 4.10 Detection of serum AFP and AFP-L3 levels by ELISA

The addition of serum samples to pre-coated enzyme-linked plates, with separate wells designated for blanks and test samples. Standard samples were concurrently assayed. The assay proceeded with the addition of corresponding enzyme reagents, followed by incubation at room temperature for 1 h. After incubation, the plates were washed and patted dry, a process repeated five times. Subsequently, a chromogenic substrate was added, inducing a colorimetric reaction for 15 min. The reaction was halted by the addition of a stop solution. The absorbance was measured at a wavelength of 450 nm, providing a quantitative evaluation of the target proteins in the serum samples.

### 4.11 Hematoxylin-eosin staining

Rat liver tissue samples measuring approximately 1.5 cm × 1.5 cm x 0.2 cm were fixed in 4% paraformaldehyde solution for 12 h. The samples were then dehydrated using a gradient alcohol series, cleared with xylene to remove ethanol, and embedded in paraffin. Tissue sections with a thickness of 3 μm were prepared and baked at 60°C for 5 h. The paraffin sections were then dewaxed in xylene and rehydrated through a graded ethanol series. After staining with hematoxylin for 10 min, the sections were washed with tap water to remove excess stain. The sections were then differentiated in hydrochloric acid alcohol solution for 5 s and washed with bluing reagent before staining with eosin solution for 3 min. Subsequently, the sections were dehydrated using a graded alcohol series, cleared with xylene to remove ethanol, and mounted with neutral resin for observation under a microscope.

### 4.12 Immunohistochemical analysis

Tissue samples were fixed in 10% buffered formalin and embedded in paraffin. Sections (5 μm) were deparaffinized and rehydrated through graded ethanol, then subjected to antigen retrieval in sodium citrate buffer (0.01 mol·L-1, pH 6.0) at 95°C for 10 min. Endogenous peroxidase activity was quenched with 3% hydrogen peroxide for 10 min to reduce background staining. Sections were blocked with goat serum for 20 min at room temperature to prevent non-specific binding of the primary antibody. The sections were then incubated with iNOS, p-PI3K, and GLUT1 primary antibody at a dilution of 1:500 overnight at 4°C. After washing, the sections were incubated with Bio-sheep anti-rabbit secondary antibody for 30 min at room temperature to amplify the signal. The sections were then incubated with streptavidin-peroxidase for 30 min to further amplify the signal and developed with 3-3-diaminobenzidine (DAB) to visualize the staining. Nuclei were counterstained with hematoxylin before dehydration, clearing, and mounting. Positive staining was visualized as brownish-yellow or brown granules.

### 4.13 Statistical analysis

Data were obtained from one of three independent experiments. Differences between groups were analyzed using one-way analysis of variance (ANOVA) followed by Dunnett’s *t*-test. A *p*-value of less than 0.05 was considered statistically significant, while a *p*-value of less than 0.01 indicated highly significant differences.

## 5 Conclusion

**FIGURE 8:**
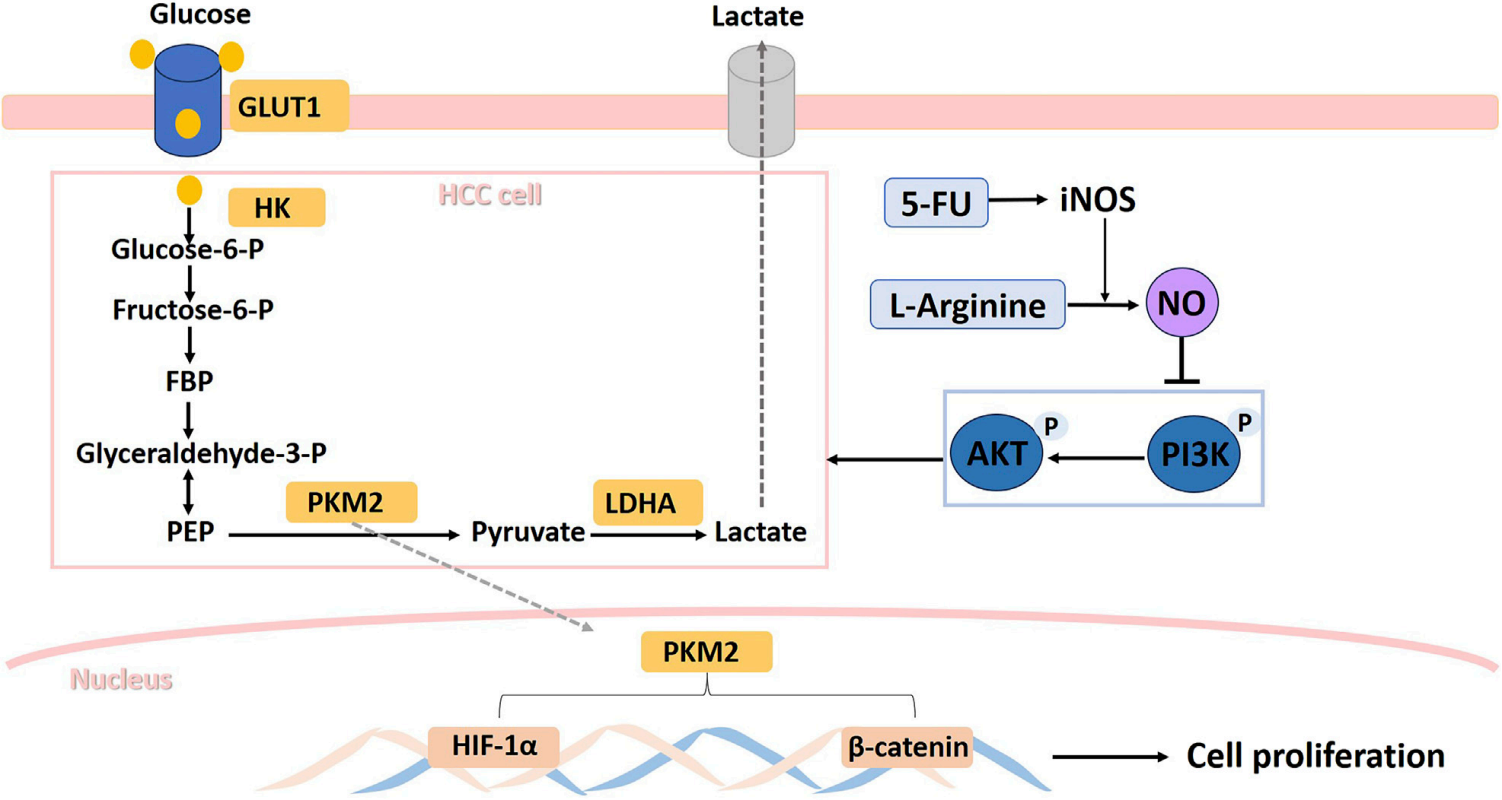
Schematic illustration of the suppressive effects of L-Arginine and 5-Fluorouracil combination on hepatocellular carcinoma cells via iNOS/NO/AKT-mediated glycolysis inhibition.

In conclusion, as shown in Figure 8, during the combined use of L-arginine and 5-FU, the increase in iNOS expression in HepG2 and SMMC7721 cells caused by 5-FU allows L-arginine to elevate NO level within HCC cells. This, in turn, inhibited the PI3K/AKT signaling pathway, which led to a reduction in the expression of proteins such as GLUT1, PKM2, and LDHA, ultimately resulting in decreased lactate production. The inhibition of these key enzymes in aerobic glycolysis also suppressed expression of nuclear transcription factors such as HIF-1α and β-catenin, ultimately inhibiting the proliferation of hepatocellular carcinoma cells. These findings suggested that the synergistic effect of L-arginine and 5-FU on glucose metabolism pathway could be a promising approach in cancer treatment.

## Data Availability

The original contributions presented in the study are included in the article/Supplementary material, further inquiries can be directed to the corresponding author.
